# Bioflocculant Production by *Virgibacillus* sp. Rob Isolated from the Bottom Sediment of Algoa Bay in the Eastern Cape, South Africa 

**DOI:** 10.3390/molecules16032431

**Published:** 2011-03-14

**Authors:** Sekelwa Cosa, Leonard V. Mabinya, Ademola O. Olaniran, Omobola O. Okoh, Kim Bernard, Shaun Deyzel, Anthony I. Okoh

**Affiliations:** 1 Applied and Environmental Microbiology Research Group (AEMREG), Department of Biochemistry and Microbiology, University of Fort Hare, Private Bag X1314, Alice 5700, South Africa; E-Mails: sekco@webmail.co.za (S.C); lmabinya@ufh.ac.za (L.V.M); 2 Division of Microbiology, University of KwaZulu Natal, Durban Westville, South Africa; E-Mail: olanirana@ukzn.ac.za (A.O.O); 3 Department of Chemistry, University of Fort Hare, Alice, South Africa; E-Mail: ookoh@ufh.ac.za (O.O.O); 4 South African Environmental Observation Network, SAEON, Elwandle Node, 18 Somerset Street, Grahamstown, South Africa, 6140; E-Mails: kimb@vims.edu (K.B); shaun@seaon@ac.za (S.D)

**Keywords:** *Virgibacillus* sp., bioflocculant, flocculating activity, polysaccharide

## Abstract

A bioflocculant-producing marine bacterium previously isolated from marine sediment of Algoa Bay was screened for flocculant production. Comparative analysis of 16S rDNA sequence identified the isolate to have 99% similarity to *Virgibacillus* sp. XQ-1 and it was deposited in the GenBank as *Virgibacillus* sp. Rob with accession number HQ537127. The bacterium produced biflocculants optimally in glucose (70.4%) and peptone (70.4%) as sole sources of carbon and nitrogen, alkaline pH (12) (74%); and the presence of Fe^2+ ^(74%). Chemical analysis of the bioflocculant revealed it to be a polysaccharide.

## 1. Introduction

Bioflocculants have recently attracted and received a considerable scientific and biotechnological attention, especially due to their biodegradability, non-toxicity, benign nature and lack of secondary pollution [1,2,3]. As examples, bioflocculants have been applied practically in various processes *i.e.,* in treatment of dye solutions, removal of humic acids, and removal of metal ions from polluted effluents [1,2,3,4,5,6]. According to Oh *et al.* [7] bioflocculants also have been used to successfully harvest *Chlorella vulgaris* from culture broth. Although a few bioflocculants have been successfully identified, general low flocculating capability, large dosage requirements and high costs are still a major concern in their development for such practical applications [1,8].

Bioflocculants mostly come from the natural secretions of bacteria and cell lysis [9]. Furthermore, they are kinds of extracellular biopolymers of macromolecular substances such as proteins, glycoproteins, polysaccharides and nucleic acids [8,10,11]. For example, *Rhodoccocus erythropolis* S-1, *Bacillus subtillis* and *Bacillus licheniforms*, are found to produce proteinaceous bioflocculants, while *Alcaligenes cupidus* KT201 was found to contain polysaccharides [12,13,14,15] and according to Lee *et al.* [16] *Arcuadendron* sp. TS-4 exhibited a glycoprotein bioflocculant. Deng *et al.* [4] reported that the effective groups for flocculation in proteinaceous bioflocculants include the amino and carboxyl groups, while polysaccharide bioflocculants are mostly based on high molecular weights. These bioflocculants produced by bacteria exhibit different flocculating activity or properties for various target compounds. Thus far, many bioflocculant-producing microorganisms (*i.e.,* bacteria, fungi, algae and yeast) have been identified [4]. Since, only a few of these bacterial strains have been used in successful industrial processes for reasons explained above and elsewhere [1,8,17,18], the need to overcome such problems becomes imperative and discovering novel efficient bioflocculants from varied environments has become an active area of research [8]. In this paper, we report on the bioflocculant-producing potential of *Virgibacillus* sp. Rob isolated from the marine environment of Algoa Bay, Eastern Cape of South Africa as part of our exploration of the South African marine environment as a source of new bioflocculant-producing organisms.

## 2. Results and Discussion

### 2.1. Screening for flocculant producing microorganism

**Figure 1:**
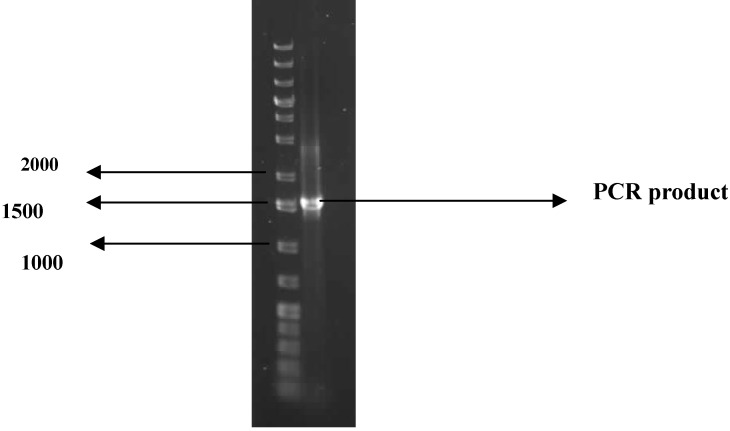
PCR product of the 16S rDNA of the test bacterium in 1% agarose gel electrophoresis. The first lane is DNA marker; while the second lane is the PCR product.

Over 300 bacteria isolates previously obtained from marine sediments of Algoa Bay in the Eastern Cape Province, South Africa were screened for bioflocculant production. One of these marine bacteria isolates exhibited appreciable ability to flocculate a kaolin suspension, with a 70.4% activity being observed. Morphologically, the bacterium colonies were round, smooth and glistening with entire margin, cream in color, mostly flat and about 1 mm in diameter. The 16S rDNA of the analysis yielded a PCR product of expected size (approx. 1.5 kb, Figure 1). Basic Local Alignment Search Tool (BLAST) analyses of the nucleotide sequence of the 16S rDNA revealed the bacterium to have 99% similarly to *Virgibacillus* sp. XQ-1 and the sequence was therefore deposited in the Genbank as *Virgibacillus* sp. Rob with accession number HQ537127.

The genus *Virgibacillus* are Gram-positive, motile, endospore-forming, irregular rod-shaped and halophilic bacteria [19], and was first proposed by Heyndrickx *et al.* [20] and later amended by Heyrman *et al.* [21]. Now the genus is comprised of sixteen recognized species [19,22]. The members of the genus are noted for the presence of *meso*-diaminopimelic acid in their cell-wall peptidoglycan, MK-7 menaquinone, diphosphatidylglycerol and phosphatidylglycerol polar lipids and the G+C content is in the 36–43 mol% range [19,22]. 

Like many halophiles, the genus *Virgibacillus* has been implicated in the production of a number of biotechnologically relevant biomolecules. A typical example of enzymes would include proteases. The enzyme proteases are found to be produced or secreted most by the genus *Virgibacillus* and are of particular interest due to their wide applications in laundry detergents, leather processing, protein recovery or solubilization, organic synthesis, meat tenderization, detergents, food industry, photography, and pharmaceuticals [23]. As an example, protease was produced by a moderately halophilic bacterium isolated from *Pla-ra,* a fermented fish product in Thailand, and identified as *Virgibacillus marismortui* NB2-1 [24]. In another study, an extracellular proteinase produced by *Virgibacillus* sp. SK37 was found very useful in fish sauce fermentation [25]. Also, *Virgibacillus pantothenticus* (MTCC 6729) isolated from fresh chicken meat samples produced a newly identified serine alkaline protease that was found useful as a stain remover additive in detergents and other bio-formulations [23]. Other studies have reported amylases [26] and antifungal compound [27] production by some *Virgibacillus* species. Although as mentioned a number of bioactive compounds have been documented to be produced by *Virgibaccillus* species, to the best of our knowledge, this is the first report implicating the *Virgibaccillus* genus in bioflocculant production. 

### 2.2. Factors affecting the bioflocculant production and flocculating activity

Constituents of the culture medium and culture conditions have been well documented to have effect on the production of bioflocculants [28,29]. Carbon and nitrogen sources have also been emphasized [2]. Therefore, the effect of culture conditions on bioflocculant production by the test bacterium was assessed and the results are as shown in Table 1. It was found that using glucose as carbon source in the medium yielded bioflocculant with the highest flocculating activity (70.4%), compared to other carbon sources. Peptone was deduced to be the preferred nitrogen source as it resulted in production of bioflocculant with the highest flocculating activity (70.4%) compared to other nitrogen sources, while in comparison to other salts iron sulphate as cation considerably induced high flocculating activity (74%). 

**Table 1:** Effects of composition of medium on the bioflocculant production by *Virgibaccilus* sp. Rob.

Carbon Source	Glucose	Sucrose	Fructose	Starch
Flocculating Activity %	70.4	-	18	5
**Nitrogen Source**	**Peptone **	**Ammonium Sulphate**	**Urea**	**Ammonium Chloride**
Flocculating Activity %	70.4	22	38	37
**Salts **	**Calcium Chloride**	**Magnesium Chloride **	**Iron Sulphate**	**Potassium Chloride **
Flocculating Activity %	70.4	-	74	-

Note: (-) denotes no flocculating activity.

As stated earlier, carbon and nitrogen sources have been well documented to have crucial effect on the bioflocculant production [2,4]. However, these may vary with different strains. As example, for *Sorangium cellulosum*, soluble starch was optimal carbon source [3]. In the case of *Klebsiella* sp., maltose and urea were found to be best carbon and nitrogen source [30]. In another study, glucose, lactose and fructose were unfavorable for the bioflocculant production by *Bacillus licheniformis*, while glutamic acid, citric acid and glycerol were favorable for its growth and bioflocculant production [15]. Also, for *Aspergillus parasiticus*, corn starch and peptone were found to produce a bioflocculant with high flocculating activity [4]. The preference by our test bacterium for glucose and peptone as sole sources of carbon and nitrogen is similar to the results obtained bioflocculant production by *Proteus mirabilis* TJ-1 [29]. Also, with *Serratiaficaria*, glucose amongst other carbon sources was suitable for the production of bioflocculant, although peptone and other nitrogen sources were not favorable [31]. In a study by Lachhwani [32], strain RDL-1 also effectively utilized glucose as the best carbon source.

It is generally accepted that the flocculation induced by bioflocculant can occur by bridging and charge neutralization [30,33]. It may also be noted that these cations may vary depending on the organism. As example, a bioflocculant from a haloalkalophilic *Bacillus* was greatly enhanced by the addition of cations like Ca^2+^, Cu^2+^, Zn^2+^, Mn^2+^, Fe^2+^, but the addition of cations like Al^3+^, Fe^3+^ and Na^+^ led to a drop in flocculating activity [11]. Also in another study, the flocculating activity of an *Aeromonas*-produced bioflocculant was increased with the addition of K^+^, Na^+^ and Ca^2+^ [34]. In our current study, the bioflocculant production by *Virgibacillus* sp. Rob was most enhanced in the presence of iron sulphate, in support of the findings of Sheng *et al.* [30] and Li *et al.* [35].

**Figure 2:**
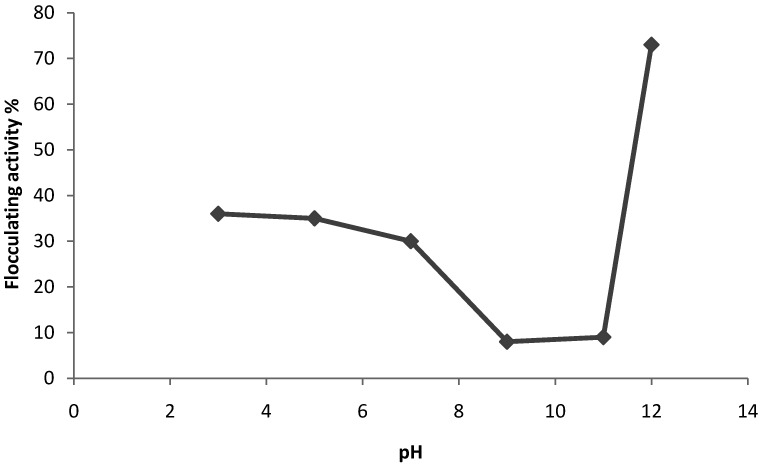
Effect of initial pH of cultivation medium on bioflocculant production by *Virgibaccilus* sp. Rob.

The pH of the culture medium may affect or influence the production of the bioflocculant [2]. The initial pH of the culture medium is said to determine the electric charge of the cells together with the oxidation potential which can affect the nutrient absorption and enzymatic reaction [29]. Consequently, the effect of initial pH on bioflocculant production by *Virgibaccilus* sp. Rob was investigated (Figure 2). A steady decrease in flocculation activities were observed from pH 3 to the lowest level of about 8% at pH 9, beyond which activity increased to an optimal level (74%) at pH 12. 

This initial pH requirement may have a different effect with different strains. For example, the strains *Streptomycetes griseus* and *Aspergillus sojae* produced flocculating substances under acidic conditions [36]. Bioflocculant production by *Rhodococcus erythropolis* was higher at alkaline pH values (8.0–9.5) [37], while in another study it showed activity at neutral pH [38]. In a study by Lachhwani [32], the flocculant production by the isolates RDL-1 and RDL-2 was greatly stimulated at slightly alkaline pH 7.5, while at acidic pH 6.0 and highly alkaline pH 10.5 it was low. In our current study, *Virgibacillus* sp. Rob preferred high alkaline pH (12.0) for optimal bioflocculant production. Li *et al.* [1] had also reported that *Bacillus licheniformis* X14 optimally produces a bioflocculant ZS-7 under alkaline conditions. 

### 2.3. Time course of bioflocculant production

**Figure 3:**
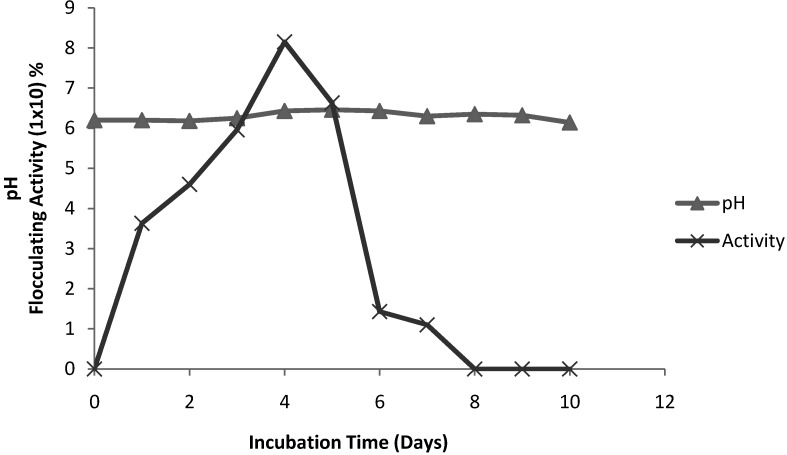
Time course of bioflocculant production *Virgibaccilus* sp. Rob.

Figure 3 shows the time course of bioflocculant production of strain *Virgibacillus* sp. Rob in relation to pH. The flocculating activity was observed to increase steadily to peak value of 81.5% in 4 days, and thereafter a steady decline in flocculating activity and from the 8^th^ day onwards flocculating activity was completely lost. Similar findings were obtained with *Streptomycetes griseus* where the flocculating activity increased with time and reached maximum activity on the fourth day [36]. Culture time for flocculant release into the medium and its activity may differ with different organisms. As an example, the peak flocculating activity of a bioflocculant produced by *Citrobacter* sp. TKF04 was obtained in 24 h of cultivation and thereafter the activity dropped [39]. In another study [17], the maximum flocculating activity of a bioflocculant produced by *Vagococcus* sp. W31 was reached after 60 h. In the case *Proteus morabilis* TJ-1 and *Bacillus licheniformis* X14, maximum cell production was reached in 24 h (1 day), while maximum flocculating activity was achieved in 48 h, and thereafter the activity decreased [1,29]. A number of factors influence bioflocculant production and thereby influence the bioflocculation process [2]. Culture time, amongst other factors, may influence the production, distribution and flocculating capabilities of the bioflocculant. The pH on the other hand remained similar (6.2–6.4) throughout the incubation period.

### 2.4. Analysis of bioflocculant composition

A small amount (0.264 g) of purified bioflocculant was recovered from a culture broth (1 L) and the freeze dried bioflocculant was white in colour. The chemical analysis of the bioflocculant showed that it contained carbohydrate (9 mg/mL) (Table 2), and no protein was detected in the compound. Hence, the bioflocculant is mainly composed of polysaccharides. Quite a number of flocculants are reported to be polysaccharides, for example, the bioflocculant produced by *Bacillus firmus* [6]. Bioflocculant HBF-3 produced by a deep sea bacterium mutant (*Halomonas* sp. V3a) was reported to be mainly polysaccharide [8] and a novel *Serratiaficaria* bioflocculant was also found to be a polysaccharide [30]. 

## 3. Experimental

### 3.1. Screening for bioflocculant-producing bacteria

Many types of marine bacteria previously isolated and characterized from bottom sediment samples of Algoa Bay in South Africa as part of the culture collections of Applied and Environmental Microbiology Research Group (AEMREG) at the University of Fort Hare, South Africa and maintained in 20% glycerol at −80 °C were screened for bioflocculant production. 

The medium and cultivation were as follows: 10 g of glucose, 1.0 g of peptone, 0.3 g of MgSO_4_. 7 H_2_O, 5 g of K_2_HPO_4_ and 0.2 g of KH_2_PO_4_ in 1 liter of filtered natural sea water. The initial pH was adjusted to 7.0 by NaOH (0.1M) and HCI (0.1M). Two loopfuls of bacterial colonies were inoculated in five milliliters of the cultivation medium and incubated with shaking at 160 rpm for five days at 28 °C [40]. At the end of incubation period, two milliliters of the fermentation broth was centrifuged (4,000 *g*, 30 min) to separate the cells. The cell free culture supernatant was analyzed for flocculating activity. 

### 3.2. Determination of flocculating activity

The flocculating activity was determined using the method previously described by Kurane *et al.* [37], in which kaolin clay was chosen as the suspended solid. Two milliliter of the culture supernatant and 3 mL of 1% CaCI_2_ were added into 100 mL of kaolin clay suspension (4 g/L) in a 100 mL flask, gently shaken and left to stand still for 5 min. The control was prepared following the same procedure but the bioflocculant was replaced by fresh broth. The absorbances of the upper phase were measured at 550 nm using a ThermoSpectronic spectrophotometer (Helios Epsilon, USA). The flocculating activity was then calculated as follows:

Flocculating rate = {(A-B)/A} × 100%

where A is the optical density of the control at 550 nm; and B is the optical density of the sample at 550 nm.

### 3.3. The effect of carbon and nitrogen sources on bioflocculant production

It has been well documented that changing the carbon and nitrogen sources highly influences bacterial growth and production of bioflocculants [30]. Hence, we assessed the effects of different carbon and nitrogen sources on bioflocculant production in the test bacterium. Carbon source candidates included glucose, sucrose, fructose and starch, while the nitrogen source candidates included ammonium sulphate, ammonium chloride (inorganic nitrogen sources) and urea (organic nitrogen sources) replacing peptone. The assessments were done in accordance with the description of Lachhwani [32]. 

### 3.4. Effects of various cations and pH on bioflocculant production

Studying the effects of metal ions, flocculant tests was conducted utilizing the procedure elaborated above, but the CaCl_2_ solution was replaced by various other metal salt solutions, and the flocculating activity was measured. Solutions of KCl, MgCl_2_ and FeSO_4_ were used as salt sources. To assess the effect of pH on flocculating activity, the initial pH of the culture medium was adjusted using HCl (0.1 M) and NaOH (0.1M) in the pH range of 3–12 [41] (specifically pH 3.0, 5.0, 7.0, 9.0, 11.0 and 12.0 were used).

### 3.5. Time course of bioflocculant production

*Composition of culture medium*: The composition of the culture medium is as described earlier in subsection 3.1 [40]. The isolate was cultured under optimal growth conditions. 

*Standardization of the inocula*: Saline solution was prepared by adding 0.45 g NaCl in 50 mL of distilled water for each of the selected isolates. Fifty milliliters of saline solution was inoculated with a loop full of colonies for each of the strains. The Optical density (OD_660 nm_) of each was measured by taking 100 µL (culture) into 1 mL of distilled water in 3 mL cuvets and readjusted if need be to give OD_660 nm_ 0.1.

*Time course assays* (modified method of Gao *et al.* [17]): The inoculated saline solution was used as seed culture for inoculum preparation. Seed culture (1% v/v) was inoculated into 150 mL of medium in 500 mL flasks (prepared in duplicates) on a rotatory shaker (160 rpm) at 28 °C. Sample was drawn at appropriate time intervals (every 24 h) for a period of 10 days and of this 2 mL was centrifuged at 4,000 *g* for 30 min and the cell free supernatant was used to determine the flocculating activity. The pH of the broth samples was also measured. 

### 3.6. Extraction and purification of bioflocculant

Purification and characterization of the bioflocculant was done following the methods described elsewhere [42,43] using media formulation based on the pre-determined optimum culture conditions. Briefly, after five days of fermentation, the culture solution was centrifuged at 4,600 rpm for 30 minutes to remove bacterial cells. One volume of distilled water was added to the upper phase and centrifuged at 4,600 rpm for 15 minutes to remove insoluble substances. To the supernatant, two volumes of ethanol were added, and the mixed solution was stirred and left to stand at 4 °C for 12 hours. The precipitate was vacuum dried to obtain the crude biopolymer. The crude product was directly dissolved in distilled water to yield a solution, to which one volume of the mixed solution of chloroform and *n*-butyl alcohol (5:2 v/v) was added. After stirring, the mixture was set aside for 12 hours at room temperature (about 20 °C). The upper phase was centrifuged at 3000 g for 15 minutes and the supernatant was concentrated at 40 °C. Thereafter two volumes of ethanol were added to recover the precipitate. 

### 3.7. Analysis/ characterization of purified bioflocculant

The protein content was measured using the Folin-Lowry method. Total sugar content was measured using phenol-sulphuric acid protocol as described by Lachhwani [22].

### 3.8. Identification of the bioflocculant-producing microorganism

*DNA extraction:* DNA extraction was conducted via the boiling method whereby 2–3 colonies were suspended in 70 µL of sterile double distilled water. The samples were heated in a water bath at 100 °C for 10 minutes, allowed to cool for 5 minutes and thereafter centrifuged at 3,000 rpm for 5 minutes. The supernatant was transferred to a clean tube and stored at 4 °C. This serves as the template in the PCR assay. 

*PCR amplification:* PCR was carried out in 50 µL reaction volume containing 2 mM MgCl_2_, 2 U Supertherm*Taq* polymerase, 150 mM of each dNTP, 0.5 mM of each primer (F1: 59-AGAGTTTGATCITGGCTCAG-39; I = inosine and primer R5: 59-ACGGITACCTTGTTACGACTT-39) and 2 µL template DNA. Primer F1 and R5 binds to base positions 7 – 26 and 1496 – 1476 of the 16S rRNA gene of *Streptomyces ambofaciens* ATCC 23877, respectively [44]. The primers in this study were used to amplify nearly full-length 16S rDNA sequences. The PCR programme used was an initial denaturation (96 °C for 2 min), 30 cycles of denaturation (96 °C for 45 s), annealing (56 °C for 30 s) and extension (72 °C for 2 min), and a ﬁnal extension (72 °C for 5 min). Gel electrophoresis of PCR products were conducted on 1% agarose gels to confirm that a fragment of the correct size had been ampliﬁed.

## 4. Conclusions

This study has shown *Virgibacillus* sp. Rob to be a potential source of new polysaccharide bioflocculant(s), the production of which could be optimized using glucose and peptone as sole carbon and nitrogen sources, as well as a pH of 12. Further characterization of the purified bioflocculant, as well as development of process conditions and practical applications for large scale production of the bioflocculant will be the subject of further research by our group.
